# Characterization and *in situ* Biodegradation Analysis of Brown Kraft Paper Coated with PHA for Potential Sustainable Packaging^§^

**DOI:** 10.17113/ftb.63.02.25.8949

**Published:** 2025-06

**Authors:** Sevakumaran Vigneswari, Mohammad Amir Huzer Mohammad Idris, Siti Nor Syairah Anis, Seeram Ramakrishna, Abdullah Al-Ashraf Amirul

**Affiliations:** 1 Institute of Climate Adaptation and Microbial Biotechnology, Universiti Malaysia Terengganu, 21030 Terengganu, Malaysia; 2 Ocular Infections and Antimicrobials Research Group, Singapore Eye Research Institute, The Academia, 20 College Road, Discovery Tower, 169856 Singapore, Singapore; 3 School of Biological Sciences, Universiti Sains Malaysia, 11800 Minden, Penang, Malaysia; 4 Center for Nanofibers and Nanotechnology, Department of Mechanical Engineering, National University of Singapore, 117581 Singapore, Singapore

**Keywords:** PHA-coated brown kraft paper, sustainable packaging material, biodegradation analysis, biodegradable films

## Abstract

**Research background:**

Biodegradable packaging is gaining immense research interest as conventional non-biodegradable food packaging has led to significant environmental pollution. In response to this, this study aims to develop biodegradable films based on polyhydroxyalkanoate (PHA) as potential food packaging material.

**Experimental approach:**

Polyhydroxyalkanoate (PHA) homopolymer, poly(3-hydroxybutyrate) [P(3HB)] and copolymer poly(3-hydroxybutyrate-co-3-hydroxyvalerate) [P(3HB-co-3HV)], known microbial biodegradable biopolymer plastics, were layered in different mass ratios using the dispersion coating technique over the brown kraft paper as a food packaging material. PHA are known to be safe, non-cytotoxic and non-genotoxic, with a remarkable ability to biodegrade in the environment. The P(3HB) and P(3HB-co-3HV) were synthesised from carbon sources of palm olein and a combination of palm olein with 1-pentanol, respectively, using *Cupriavidus malaysiensis* USMAA2-4_ABH16_, a transformant bacterial strain with acquired lipase genes.

**Results and conclusions:**

Contact angle analysis indicated that brown kraft paper coated with P(3HB-co-3HV) had a higher contact angle than uncoated brown kraft paper and paper coated with P(3HB). The biodegradation analysis of brown kraft paper coated with P(3HB) showed that it degraded 100 % within 9 days compared to all samples of brown kraft paper coated with P(3HB-co-3HV), which were completely degraded by day 12.

**Novelty and scientific contribution:**

The results show that brown kraft paper coated with P(3HB-co-3HV) was more hydrophobic than uncoated and P(3HB)-coated brown kraft paper. This study encourages further investigations of brown kraft paper coated with PHA to develop biodegradable food packaging, paving the way for a sustainable alternative to non-biodegradable packaging material.

## INTRODUCTION

Large quantities of plastic debris accumulate in the environment and in landfills with negative environmental impact (*1*). The plastic crisis is worsening day by day as global plastic production exceeds 450 million tonnes annually (*2*). Plastic waste generation has risen to 360 million metric tonnes per year and global plastic pollution is expected to double by 2040 (*3*). Synthetic plastics are often mixed with various additives such as plasticisers (phthalates in PVC), stabilisers (UV stabilisers in outdoor plastics), fillers (calcium carbonate in polypropylene), colourants and antimicrobial additives (metal nanoparticles) to improve their properties, such as durability, flexibility and resistance to degradation (*4*). These additives can have an impact on the environment, especially when plastics break down or are disposed of improperly (*5*, *6*). These mostly toxic additives are known endocrine disruptors and can leach into food, water and the environment, leading to long-term health risks (*7*). This has led to the search for suitable substitutes for synthetic plastic materials, so that biodegradable packaging has gained greater visibility, especially in the food industry (*8*). As a result, biodegradable plastics have been favoured as an alternative and their production has increased considerably over the decades (*9*).

There are various types of biodegradable plastics such as polylactic acid (PLA), starch-based plastics, polycaprolactone (PCL), cellulose-based plastics and polyhydroxyalkanoates (PHA). PHA gained global attention because they are biodegradable in the environment without leaving microplastic residues, as well as their non-genotoxicity and non-cytotoxicity (*10*). PHA is a natural polymer that accumulates under nutrient limitation but with excess carbon sources. Common PHA copolymers include poly(3-hydroxybutyrate-co-3-hydroxyvalerate) [P(3HB-co-3HV)] and poly(3-hydroxybutyrate-co-4-hydroxybutyrate) [P(3HB-co-4HB)], which are produced by bacterial fermentation processes (*11*, *12*).

The PHA-based material is often used as a packaging material because PHA-based polymers serve as biodegradable barrier coating materials (*13*, *14*). In a previous study, a biocomposite of P(3HB) and cellulose was produced as a biodegradable packaging material; however, its properties were improved only when a certain amount of PHB was added to the cellulose paper (*13*). Moreover, research on food packaging focusing on biodegradability was the main objective. In the aquatic environment, the percentage of PHA degradation is higher (*11*, *15*). This is due to the presence of PHA depolymerase, which is produced by PHA-degrading microorganisms and hydrolyses water-insoluble PHA (*16*). In addition, the percentage of degradation depends on several factors: temperature, pH, nutrient supply, microbial population, and the composition and crystallinity of PHA (*17*, *18*).

Nowadays, coated brown kraft paper is widely used as food packaging. The versatility, low-cost, easy production and availability make the coated brown kraft paper suitable for use in various applications, such as packaging, printing, writing and household products (*19*, *20*). Nevertheless, fossil-based coatings are commonly used on the brown kraft paper to improve barrier properties, moisture resistance, water resistance and durability. These coatings are typically derived from petroleum-based polymers such as polyethylene, polypropylene, polyvinyl chloride (PVC), waxes and polyvinylidene chloride (PVDC). However, concerns about environmental impact and microplastic pollution have led to increased research into sustainable alternatives (*20*, *21*) that are biodegradable and generally not harmful when discarded in the environment.

In this study, brown kraft paper with PHA, namely P(3HB) and P(3HB-co-3HV), was prepared using the dispersion coating technique. The characterization and percentage of degradation of brown kraft paper coated with PHA in a natural environment (lake) was analysed. This study was conducted to produce biodegradable PHA-coated brown kraft paper for packaging applications that require a moisture or grease barrier and to provide strength to the brown kraft paper for various applications, especially in the food industry.

## MATERIALS AND METHODS

### Production of PHA

The transformant bacterial strain with acquired lipase genes, *Cupriavidus malaysiensis* USMAA2-4_ABH16_, previously described (*18*), was used in this study for the biosynthesis of P(3HB-co-3HV) (*22*). The wild strain of *Cupriavidus malaysiensis* was isolated from Lake Kulim, Kulim, Malaysia, and analysed for PHA production as previously described (*23*, *24*). In pure batch fermentation at a room temperature of 30 °C and a working volume of 15 L in the bioreactor (Biostat-D100; B Braun, Melsungen, Germany), P(3HB-co-3HV) was accumulated in 84 h. An inoculum size of bacterial cells of approx. 0.06 g/L was cultured in a mineral salt medium (MSM) with the addition of carbon sources of 7.05 g/L sterilised palm olein, 1.5 g/L sterile 1-pentanol and 1.5 g/L ammonium sulphate.

**Fig. S1:**
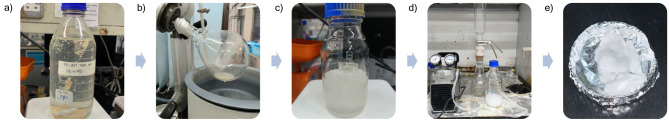
Diagram showing the steps in polymer extraction: a) disruption of freeze-dried cells and dissolution of polyhydroxyalkanoate (PHA) with chloroform, b) concentration of polymer solution in a rotary evaporator, c) precipitation of PHA with chilled methanol, d) recovery of PHA with a vacuum pump, and d) recovery of crude PHA

The observation of dissolved oxygen spike was based on dissolved oxygen-stat mode (DO-stat mode). The P(3HB) was biosynthesised in shake flasks and incubated at 30 °C and 200 rpm for 3 days to accumulate PHA polymer. A working volume of 50 mL was used with 0.06 g/L of inoculum, transferred into 50 mL of MSM that contained 7.05 g/L sterilized palm olein. The cells that accumulated PHA were harvested and freeze-dried. PHA was extracted using freeze-dried cells stirred in chloroform at a ratio of 1:200 (*m*/*V*) for 24 h at room temperature. The filtrate was concentrated in a rotary evaporator, precipitated in chilled methanol and filtered using a 0.45 μm polytetrafluoroethylene (PTFE) membrane before left to dry in a desiccator, as shown in Fig. S1 (*23*).

### Preparation of brown kraft paper coated with PHA

Brown kraft paper with dimensions of 2.0 cm×2.0 cm was prepared. Samples with mass ratios of substrate (paper) to pure polymer of 100:0, 90:10, 80:20, 70:30, 60:40 and 50:50 were used. The volume of chloroform varied from 1.0 to 1.5 mL, depending on the mass of pure polymer dissolved in chloroform. The chloroform was evaporated completely before further testing.

The dispersion coating technique was applied in this work, using a 2.0 cm×2.0 cm mould. The paper was placed in the mould and the coating solution was dispersed over the surface of the paper by tapping several times to spread the coating solution over the entire surface. The coated papers were left to dry for 3 days.

### Characterization of brown kraft paper coated with PHA

#### Mass and thickness of brown kraft paper coated with PHA

The thickness of brown kraft paper coated with PHA was measured using dial thickness gauge (SM-112; Teclockat, Nagano, Japan) at five different locations on the same sample.

The mass of PHA coating (g) was calculated using the equation below:


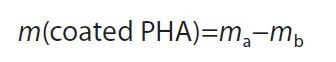
 /1/

where *m*_a_ is the mass (g) of brown kraft paper coated with PHA and *m*_b_ is the mass (g) of brown kraft paper.

#### Density of brown kraft paper coated with PHA

Based on the obtained average thickness (*l*) of PHA-coated brown kraft paper and its diameter, the density of the coated paper was calculated using the following equations:


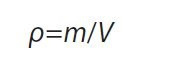
 /2/


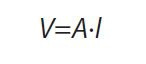
 /3/

#### Water contact angle measurement

Water contact angle was measured by dispersing water droplets on the surface of the samples using a computer graphic software ImageJ v. 13.0 (*25*, *26*). Briefly, the smartphone with camera lens (iPhone 7 plus, front facing camera 12 MP) was placed on the smartphone holder with the camera application opened and focused on the sample. The sample was placed on a microscope slide at the end of a work bench with a distance of 1–2 cm from the smartphone camera. The angle and the height of the sample were adjusted to ensure that the surface of the sample was horizontal when viewed through the phone (*27*). A small drop of approx. 10 µL water was dropped onto the surface of the sample. The images were taken using the smartphone camera with burst mode and uploaded to ImageJ software to calculate the contact angle.

### Degradation of brown kraft paper coated with PHA

The percentage of degradation of brown kraft paper coated with PHA was observed in Lake Kulim, where a greater number of microorganisms that can degrade or synthesise PHA are present (*16*). Suitable sites were selected to place the samples and observe the degradation. Tasik Fajar, Universiti Sains Malaysia (5°21’13”N 100°18’01”E) was chosen as the location for this study as it has been used in previous studies as a model for freshwater environment to evaluate the degradation of PHA and PHA composites (*16*, *28*).

The immersion test was performed with triplicate samples. The samples at a size of 2.0 cm×2.0 cm were immersed in the lake and monitored once every 3 days for a total of 12 days. Temperature, pH and dissolved oxygen were also recorded once every day for a total of 12 days. The samples were collected during the observation intervals and then wiped, washed several times with distilled water and dried at room temperature. The initial mass and the final mass after degradation were recorded. The percentage of degradation was calculated using the following equation:


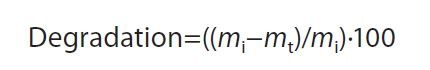
 /4/

where *m*_i_ is the initial mass of the sample (g) and *m*_t_ is the final mass of the sample after each degradation (g).

### Statistical analysis

The qualitative results were presented as mean value and standard deviation (S.D). All values at p<0.05 were considered significant. For statistical analyses, ANOVA and Tukey’s honestly significant difference (HSD) test were used with the IBM SPSS Statistics v. 29.0 software (*29*).

## RESULTS AND DISCUSSION

### Production and characterization of brown kraft paper coated with PHA

**Fig. S2:**
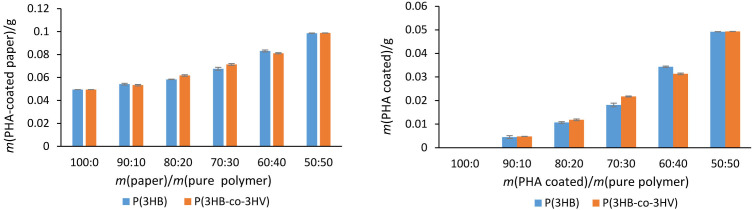
The mass of: a) paper coated with polyhydroxyalkanoate (PHA) and b) pure PHA polymer

With increasing mass ratio of substrate (paper) to pure polymers in the order 50:50>60:40>70:30>80:20>90:10>0:100 (Fig. S2a), the brown kraft papers coated with PHA tend to wrinkle at the edges. This is probably due to the coating of polymers on the paper surface, which caused the edges of the paper to wrinkle. The P(3HB-co-3HV)-coated brown kraft paper had a smooth surface and was less brittle than paper coated with P(3HB), which had a rough surface and was more brittle. This is due to the low crystallinity of the copolymer, resulting from the addition of 3HV monomer to the brittle structure of P(3HB) (*30*). The formation of bubbles on the surfaces of both PHA-coated papers was due to the presence of air when the coating solution was transferred to the paper surfaces.

The pure polymers for both the homopolymer of P(3HB) and the copolymer of P(3HB-co-3HV) were used in different mass ratios of substrate (paper)to pure polymers of 100:0, 90:10, 80:20, 70:30, 60:40 and 50:50 (Fig. S2b). With the increase of the substrate (paper) mass in sample mass ratio, differences in the morphology of papers coated with PHA, such as the surface roughness of the coated paper, were observed. The surface roughness of the coated paper increased proportionally from a sample mass ratio of 80:20 to 50:50. Higher sample mass ratios resulted in the formation of wrinkles on the edge of the PHA-coated brown kraft paper. P(3HB)-coated brown kraft paper had a brittle polymer layer compared to P(3HB-co-3HV)-coated brown kraft paper. At a sample mass ratio of 50:50, the mass of P(3HB)-coated brown kraft paper and P(3HB-co-3HV)-coated brown kraft paper was 0.0986 and 0.0988 g, respectively. At the mass ratio of 100:0, the mass of the paper was 0.0495 g, as no polymers were coated on the paper. The samples were produced using the dispersion coating method, and all samples of the papers coated with both P(3HB) and P(3HB-co-3HV) were obtained. This could be a result of the increase in the mass of the pure polymer used to prepare the coating solution and also to coat the substrate. At a sample mass ratio of 50:50, the mass of both P(3HB) and P(3HB-co-3HV) coated on the substrate was higher on average (0.0492 and 0.0493 g, respectively).

**Fig. 1:**
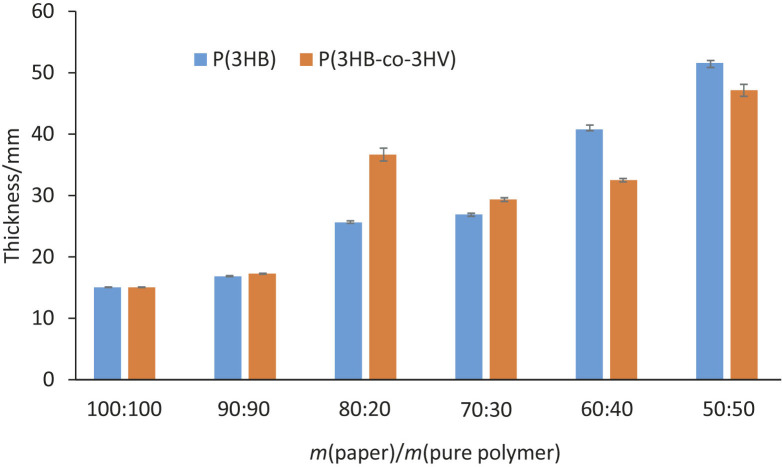
The thickness of brown kraft paper coated with P(3HB) and P(3HB-co-3HV) for different sample mass ratios

Similar trends were observed for film thickness, as shown in Fig. 1. The thickness of the PHA-coated brown kraft papers increased with sample mass ratio of substrate (paper) to pure polymers in the following order: 100:0>90:10>80:20>70:30>60:40>50:50. This is due to the increased use of pure polymer in the coating, which led to an increase in the thickness of the polymer layer. The thickness of PHA-coated brown kraft paper increased up to a sample mass ratio of 50:50, with a thickness of P(3HB) and P(3HB-co-3HV) of 0.52 and 0.47 mm, respectively. With a sample mass ratio of 100:0, the thickness of the paper was 0.15 mm. Compared to the sample mass ratio of 90:10, the thickness of the papers coated with either P(3HB) or P(3HB-co-3HV) was 0.17 mm. As the thickness of the PHA-coated brown kraft papers increased, the PHA-coated brown kraft papers became thicker. Finally, thicker PHA-coated brown kraft paper had a higher elongation at break (*30*, *31*).

### Physical and mechanical properties and biodegradability of brown kraft paper coated with PHA

#### Density analysis

The density of brown kraft papers coated with P(3HB) and P(3HB-co-3HV), and paper without polymers (sample mass ratio of 100:0) is shown in Table 1.

**Table 1:** The density of brown kraft paper coated with P(3HB) and P(3HB-co-3HV)

	*ρ*(PHA-coated paper)/(g/cm^3^)	
*ζ*(sample)	P(3HB)	P(3HB-co-3HV)
100:0	0.82	0.82
90:10	0.82	0.78
80:20	0.59	0.42
70:30	0.62	0.61
60:40	0.51	0.63
50:50	0.48	0.53

*ζ*=*m*(paper):*m*(pure polymer). Density was calculated using the average thickness obtained

It was found that the densities of the brown kraft paper without polymers and PHA-coated paper was lower than that of water (0.99 g/cm^3^). This indicates that the P(3HB)-coated and P(3HB-co-3HV)-coated brown kraft papers would float on the water surface (*32*). The density of kraft paper coated with P(3HB) and P(3HB-co-3HV) decreased with the increase of polymer mass in the sample mass ratio. In addition, as the thickness of the two coated papers increased, their volume also increased. This resulted in a decrease in the density of both types of coated papers due to the relationship between the density and volume of PHA-coated papers, which was inversely proportional (*32*).

As reported by Godbole *et al.* (*33*), if the density of the material is higher than the density of water (0.99 g/cm^3^), the material is likely to sink to the bottom of the water. Compared to the density of uncoated brown kraft paper and PHA-coated brown kraft paper, which were lower than the density of water, all samples will float on the surfaces of the water.

#### Water contact angle analysis

The contact angles measured on the surface of papers coated with P(3HB) and P(3HB-co-3HV) and the uncoated brown kraft paper are shown in Table 2.

**Table 2:** The contact angle (*θ*/°) of brown kraft paper coated with P(3HB) and P(3HB-co-3HV)

	*θ*/°	
*ζ*(sample)	P(3HB)	P(3HB-co-3HV)
100:0	(67.8±2.0)^a^	(67.8±2.0)^a,b^
90:10	(63.5±3.5)^a^	(64.2±2.7)^a^
80:20	(80.9±4.8)^b^	(85.0±11.62^b,c^
70:30	(112.1±2.8)^c^	(114.8±7.6)^d^
60:40	(88.1±2.8)^b^	(88.1±2.8)^c^
50:50	(112.9±1.3)^c^	(112.9±7.6)^d^

*ζ*=*m*(paper):*m*(pure polymer). Data are represented as mean value±S.D. (*N*=3). The Tukey's HSD test indicates that different letters denote significant differences within the concentration at the p<0.05 level

At a sample mass ratio of 100:0, which was paper only, the contact angle was 67.8°, the smallest angle recorded compared to the other PHA-coated brown kraft papers. At a sample mass ratio of 50:50, the contact angle of P(3HB)-coated brown kraft paper was larger (112.9°) than the contact angles of the other samples. At a sample mass ratio of 70:30, the contact angles of P(3HB)-coated and P(3HB-co-3HV)-coated brown kraft paper were larger (112.1 and 114.8°, respectively) than the contact angles of the other samples. The large contact angle was similar to that of the paper samples coated with P(3HB) and P(3HB-co-3HV) at a 50:50 mass ratio. This could be attributed to the surface formed during the moulding of the PHA-coated brown kraft paper. In general, the contact angle of brown kraft papers increased when the mass of polymer coatings increased in the sample mass ratio. This shows that the hydrophobicity of the P(3HB)-coated and P(3HB-co-3HV)-coated brown kraft papers increased, too. The hydrophobicity of the PHA-coated brown kraft was generally higher than that of the uncoated brown kraft paper (sample mass ratio of 100:0) due to the high interfacial tension of the water molecules with the surface of the samples (*34*, *35*). Due to the higher hydrophobicity of the PHA-coated brown kraft papers and the pure PHA polymers, the water resistance of these samples increased (*20*). This prevents PHA-coated brown kraft papers from getting wet easily when used in food packaging.

This was previously reported by Petersen *et al.* (*36*), who suggested that PHA had hydrophobicity or water-insoluble properties. P(3HB-co-3HV) had a higher contact angle than P(3HB)-coated and P(3HB-co-3HV)-coated brown kraft papers and pure polymers. This is due to the presence of the additional functional group in the chemical structure of P(3HB-co-3HV), which leads to an increase in the hydrophobicity of the samples (*37*). However, the contact angle could vary depending on the surfaces of the samples. Flat surfaces would likely have a good contact angle, while rough surfaces would likely have an accurate contact angle.

#### Biodegradability analysis

**Fig. S3:**
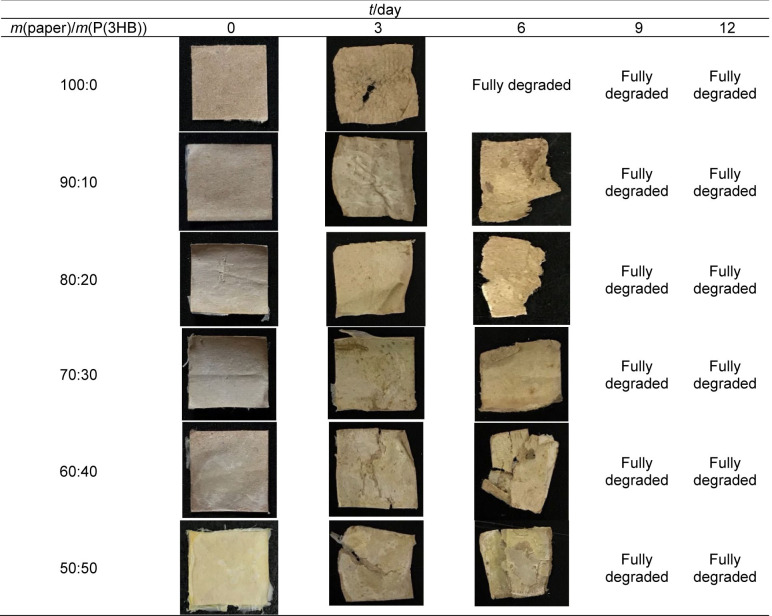
Physical changes in brown kraft paper coated with P(3HB) throughout the degradation period

**Fig. S4:**
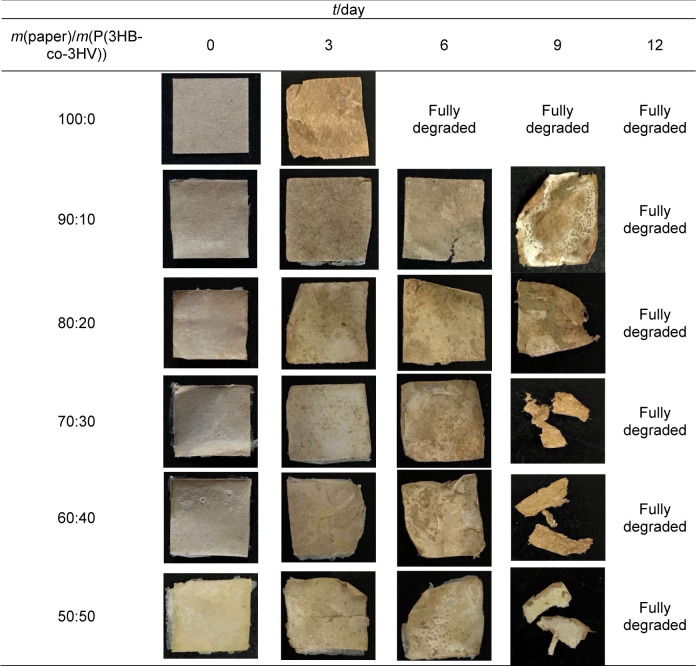
Physical changes in brown kraft paper coated with P(3HB-co-3HV) throughout the degradation period

The degradation of uncoated and polymer-coated brown kraft paper was analysed by immersing the samples in the lake (*38*). The physical changes of uncoated brown kraft paper and the two coated papers throughout degradation studies are shown in Fig. S3 and Fig. S4, respectively. The ability of PHA homopolymers, such as P(3HB) and copolymer P(3HB-co-3HV), to be degraded anaerobically is well known and leads to their various applications in agriculture as biodegradable plastics and in the food industry as biodegradable food packaging (*36*). In response to environmental factors, such as temperature, pH and biological activity, the polymer was degraded, which correlated with the changes in its chemical and physical configuration (*20*, *39*). The degradation rate or the percentage of degradation also correlated with other factors such as biological activity, which included the presence of PHA depolymerase that was released from PHA-degrading microorganisms, the crystallinity, molecular mass and the types of functional groups that were present in the polymer structure (*40*).

Based on the conducted study, the degradation of brown kraft papers coated with P(3HB) and P(3HB-co-3HV) in the freshwater environment (lake) was done in the span of 12 days with four sampling points. The temperature of the lake was recorded between 28 and 30 °C. The percentage of degradation was evaluated by mass loss during the degradation period and also by the observation of physical changes on the surface of the samples (*15*). The degraded samples were more fragile and turned into tiny and thinner masses of yellowish colour as samples were immersed longer in the lake. It is observed that at the end of the sample point, which was on day 12, all samples were fully degraded.

**Fig. 2:**
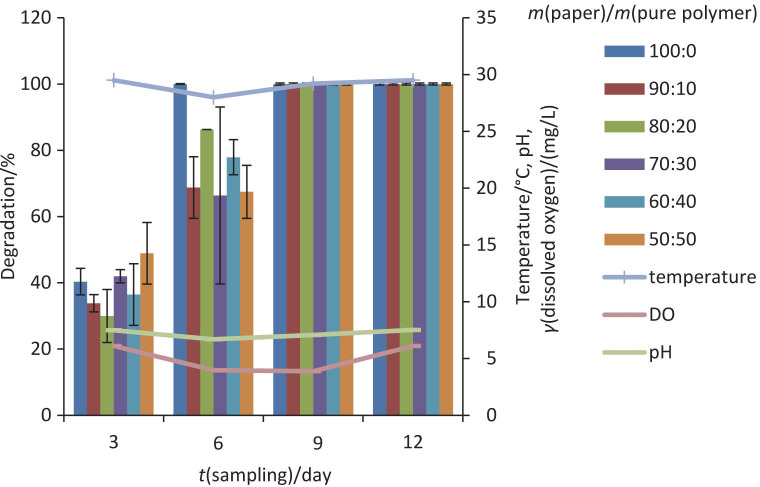
The percentage of degradation of brown kraft paper coated with P(3HB) on respective sampling days. Results are based on three replicates. Sample ratio=mass of paper/mass of pure polymer. Samples 70:30 (day 3), and 70:30, 60:40 and 50:50 (day 6) are based on two replicates as the rest of the samples could not be retrieved from the environment (lake). DO=dissolved oxygen

The results in Fig. 2 show that the percentage of degradation of all samples at all ratios of both PHA-coated brown kraft papers increased with time. As for the degradation on day 6, the uncoated brown kraft paper was fully degraded. The degradation pattern of the rest of the samples was in the following order: 90:10>80:20>70:30>60:40>50:50 with complete degradation of the sample coated with P(3HB) by day 9.

**Fig. 3:**
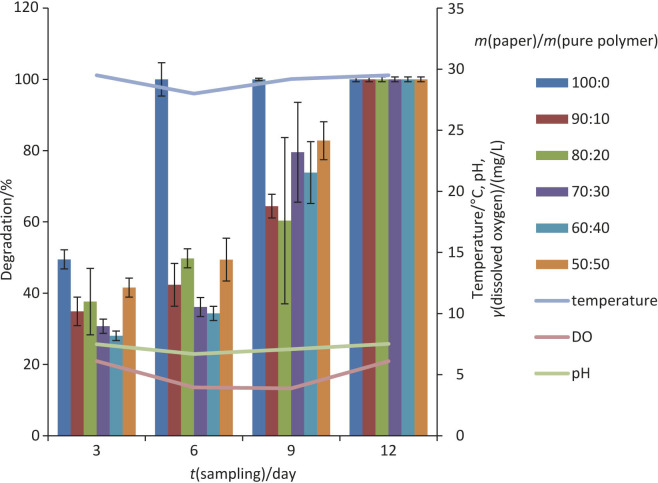
The degradation of brown kraft paper coated with P(3HB-co-3HV) on respective sampling days. Results are based on three replicates. *ζ*(sample)=*m*(paper):*m*(pure polymer). Samples 100:0 and 60:40 (day 3), and samples 90:10, 80:20, 50:50 (day 9) are based on two replicates as the rest of the samples could not be retrieved from the environment (lake). DO=dissolved oxygen

Fig. 3 shows that, as with the sample of P(3HB-co-3HV) and similarly with P(3HB)-coated brown kraft paper, all sample mass ratios were fully degraded by day 12. The paper without polymers (sample mass ratio of 100:0) had the highest degradation rate. The paper used was a recyclable brown kraft paper that easily dissolved and broke down into smaller particles in waterbodies in a short time. Similar disintegration pattern was observed elsewhere (*41*). It can be deduced that the degradation rate of brown kraft paper coated with P(3HB) and P(3HB-co-3HV) depends on the presence of a PHA layer on the surface, which is more hydrophobic than the paper.

This was reported by Rastogi and Samyn (*20*), who found that the polymer layer was hydrophobic and water resistant. The degradation rate depended on the biological activity of the PHA depolymerase secreted by PHA-degrading microorganisms, the crystallinity, the molecular mass and also the type of functional groups present in the polymer structure that caused PHA depolymerization and polymer splitting (*40*, *42*). Biodegradation of the samples is caused by an enzymatic reaction of the PHA-degrading microorganisms, which convert the polymers into carbon dioxide and water under aerobic conditions. The degradation products were used by the PHA-degrading microorganisms as the primary source of energy and nutrients (*15*, *43*).

With increasing mass fraction of polymers in the samples, the percentage of degradation decreased slightly due to the increase in the molecular mass and crystallinity of the used polymer (*40*). The P(3HB)-coated paper in the lake degraded faster than the P(3HB-co-3HV)-coated paper; the papers were completely degraded after 6 and 12 days, respectively.

A previous study showed that a recycled paper took 10 days to degrade 100 % in a lake (*44*). Furthermore, previous studies have shown that, in addition to the types of polymer used, the temperature and the mineral component of the water also determine the polymer degradation rates in a pool of water (*37*, *45*). Previous studies have also shown that P(3HB)- and P(3HB-co-3HV)-based biofibre composites produced using different techniques degraded completely in 6 and 8 weeks, respectively (*16*). The P(3HB-co-4HB) copolymer, which was produced using a salt leaching technique, also degraded faster than the solvent-cast films (*28*). It can be deduced that different production processes contribute to the degradation of the samples. This is probably due to the high microbial activity and the hydrolysis process in the lake (*40*).

The presence of water promotes hydrolytic activity through the release of enzymes by various microorganisms in the lake. The physical forces and the number of collisions with the samples by the water flow would probably increase the rate of degradation, resulting in the damage to the sample surfaces (*16*). The physical properties of the polymer, such as composition and crystallinity, also play a role in the degradation rate of the polymer. As reported by Antunes *et al.* (*30*), the copolymer P(3HB-co-3HV) with a higher 3HV content would likely increase the degradation rate.

Environmental factors, such as temperature, pH and dissolved oxygen, also act as factors of degradation. An increase in temperature would likely increase the rate of degradation of the samples. Higher temperatures would cause thermal depolymerization and conformational changes in the polymers (*46*). Both hydroxide ion and hydrogen ion from the water molecule catalyse the hydrolysis of the polymer in the lake (*47*). However, there were no significant changes in temperature, which was measured at 28–30.5 °C for 12 days throughout the degradation study. The pH was 6.69–7.52. The dissolved oxygen was 3.96–7.11. It has been previously reported that lower dissolved oxygen leads to a lower oxygen uptake by the PHA-degrading microorganisms responsible for degradation due to the lower oxygen content in the lake (*46*).

Because of their favourable properties, PHAs are increasingly used for disposable packaging, including antimicrobial food containers (*48*). These PHA-based materials not only extend the shelf-life of packaged foods, but also contribute to reducing the environmental impact (*49*). Additionally, PHAs offer a distinct advantage over other biobased polymers such as polylactic acid (PLA). While PLA can be used as a bioplastic, it does not biodegrade effectively in aquatic environments, making PHAs a more favourable option where degradation in water is an issue (*50*, *51*).

## CONCLUSIONS

In this study, the preparation of brown kraft papers coated with poly(3-hydroxybutyrate) [P(3HB)] and poly(3-hydroxybutyrate-co-3-hydroxyvalerate) [P(3HB-co-3HV)] with the paper mass to pure polymer mass ratios of 100:0, 90:10, 80:20, 70:30, 60:40 and 50:50, and their biodegradation in the Lake Kulim were investigated. The density of paper without polymers and brown kraft paper coated with polyhydroxyalkanoate (PHA) was lower than that of water (0.99 g/cm^3^), which indicates that all samples floated on the surface of the water and degraded. Biodegradation analysis showed that brown kraft paper coated with P(3HB) completely degraded within 9 days, while the paper coated with P(3HB-co-3HV) was completely degraded by day 12. Uncoated brown kraft paper, on the other hand, completely degraded by day 6.

These results encourage further investigation of brown kraft paper coated with P(3HB) to potentially develop a new biodegradable food packaging that could serve as a sustainable alternative to non-biodegradable packaging material. Further development should ensure environmental sustainability with PHA as a biodegradable food packaging.
